# Humoral immunoreactivity to gliadin and to tissue transglutaminase is present in some patients with multiple myeloma

**DOI:** 10.1186/1471-2172-9-22

**Published:** 2008-05-28

**Authors:** Zorica Juranic, Jelena Radic, Aleksandra Konic-Ristic, Svetislav Jelic, Biljana Mihaljevic, Ivan Stankovic, Suzana Matkovic, Irina Besu, Dušica Gavrilović

**Affiliations:** 1 Institute for Oncology and Radiology of Serbia, Pasterova 14, Belgrade, Serbia; 2 Faculty of Pharmacy, University of Belgrade, Belgrade, Serbia; 3 School of Medicine, University of Belgrade, Belgrade, Serbia

## Abstract

**Background:**

Multiple myeloma (MM) is a clonal B-cell disorder with many immunological disturbances. The aim of this work was to assess whether some of food antigens contribute to the imbalance of immune response by screening the sera of MM patients for their immunoreactivity to food constituent gliadin, to tissue transglutaminase-2 (tTG-2) and to Ro/SSA antigen.

Sera from 61 patients with MM in various stages of disease, before, or after some cycles of conventional therapy were analyzed by commercial Binding Site ELISA tests. The control group consisted of 50 healthy volunteers. Statistical analysis of data obtained was performed by Mann Whitney Test.

**Results:**

The higher serum IgA immunoreactivity to gliadin was found in 14/56 patients and in one of control people. The enhanced serum IgG immunoreactivity to gliadin was found in only two of tested patients and in two controls. The enhanced IgA immunoreactivity to tTG-2 was found in 10/49 patients' sera, while 4/45 patients had higher serum IgG immunoreactivity. The enhanced serum IgG immunoreactivity to RoSSÀ antigen was found in 9/47 analyzed MM patients' sera. Statistical analysis of data obtained revealed that only the levels of anti-tTG-2 IgA immunoreactivity in patients with MM were significantly higher than these obtained in healthy controls (P < 0.02)

**Conclusion:**

Data obtained showed the existence of the enhanced serum immunoreactivity to gliadin, tTG-2 and Ro/SSA antigens in some patients with MM. These at least partially could contribute to the immunological imbalance frequently found in this disease.

## Background

Multiple myeloma (MM) is a clonal B-cell disorder which diagnosis comprise the examination of bone marrow for plasma cell infiltration, detection and quantification of monoclonal protein "M" component in the serum or urine, and evidence of end-organ damage (hypercalcemia, renal insufficiency, anemia or bone lesions). Many of the laboratory parameters contribute to myeloma diagnosis due to plenty of immunological disturbances [1]. It was shown that the antibodies contained in M component have various specificity to: some proteins, double-stranded DNA, several antibiotics [2], and sometimes to gliadin (and/or calreticulin?) [3].

As the enhanced levels of the serum antibodies to gliadin are found in patients with celiac disease, as well as of antibodies to transglutaminase-2 (TTG-2) [4,5], to calreticulin [6,7] and Ro/SSA antigen [8], the aim of this work was the screening of MM patients' sera for their immunoreactivity to food constituent gliadin, and to autoantigens: tissue transglutaminase-2 (tTG-2) and Ro/SSA antigen, in order to assess whether immunoreactivity to mentioned antigens at least partially contributes to the immunological imbalance in multiple myeloma.

## Methods

### Patients

Sera from 61 patients with MM in various stages of disease, before or after some cycles of conventional therapy, were analyzed for immunity to gliadin, tissue TTG-2, and Ro/SSA antigen. Determination of serum IgA and IgG immunoreactivity to gliadin (IU/ml), to tTG-2 (IU/ml), or to Ro/SSA (IU/ml), was done by diagnostic, commercial ELISA (Binding Site) tests. Briefly, 100 μl of diluted (1:100) human sera, commercial controls and calibrators were dispensed in appropriate wells of the plates provided in the kit. During the first incubation, autoantibodies recognizing the antigen bind to it and all unbound proteins were removed by washings. After that, purified peroxidase labeled rabbit anti human IgA or IgG conjugate (100 μl) which binds to the captured human autoantibodies was added, and the excess unbound conjugate is removed by washings. The conjugate was treated with TMB (3,3,5,5-tetramethylbenzidine). The reaction was stopped by the addition of phosphoric acid. The concentration of autoantibodies was measured on ELISA reader at 450 nm. Cut offs for each test was evaluated as the mean X+2 SD.

The control group consisted of 50 healthy volunteers (age range was 27–58 years, 26 were female).

Statistical analysis of data obtained was performed by Mann Whitney Test.

Experimental research that is reported in the manuscript has been performed with the approval of the ethics committee of Institute of Oncology and Radiology of Serbia.

## Results

Cut off values of anti-gliadin reactivity obtained analyzing 50 healthy sera were 3.46 IU/ml for IgA and 5.83 IU/ml for IgG.

The elevated serum IgA immunoreactivity to gliadin was found in 14/56 patients and in one of controls. From these patients, 4 were with IgA myeloma and 4 were with IgG myeloma, while 6 were without M component in their sera.

Statistical analysis of data obtained revealed that the level of anti-gliadin IgA immunoreactivity for patients with MM was not significantly differ than that for controls (P = 0.052).

**Figure 1:**
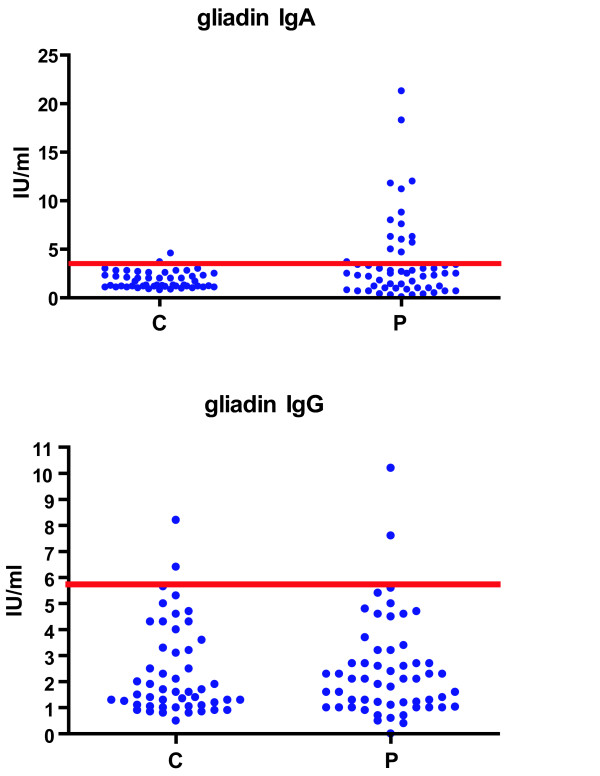
The antigliadin serum IgA and IgG immunoreactivity of healthy controls and of patients with myeloma.

Surprisingly, the elevated IgG immunoreactivity to gliadin was found only in two of tested MM patients sera and in two of control people (Fig 1.) and both patients were with IgG myeloma.

Cut off values of anti-tTg reactivity obtained analyzing 50 healthy sera were 1.86 IU/ml for IgA and 6.17 IU/ml for IgG.

**Figure 2:**
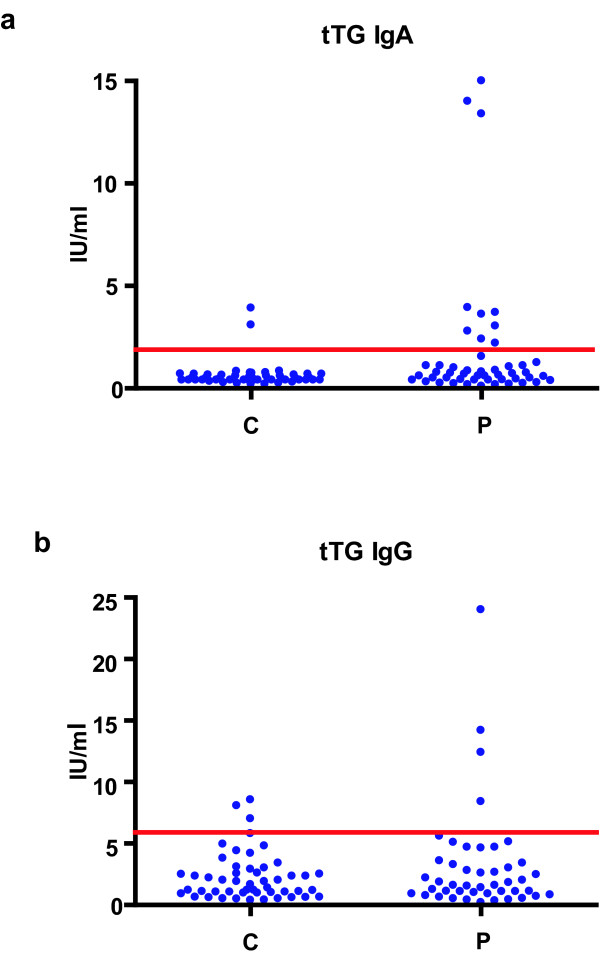
The anti tTG serum IgA and IgG immunoreactivity of healthy controls and of patients with myeloma.

As seen on Fig. 2.a. higher than cut off of IgA immunoreactivity to tTG was found in 10/49 patients and 2 of controls. From 10 anti-tTg IgA positive patients 4 were with IgA and 4 were with IgG myeloma, while 2 were without M component in their sera. Statistical analysis of data obtained revealed that the level of anti-tTG-2 IgA immunoreactivity in patients with myeloma was significantly higher than that obtained in healthy controls (P < 0.02).

Sera from 4/45 patients and 3 controls had higher than cut off value for IgG immunoreactivity (Fig. 2.b). All 4 anti-tTg IgG positive patients were with IgG MM.

**Figure 3:**
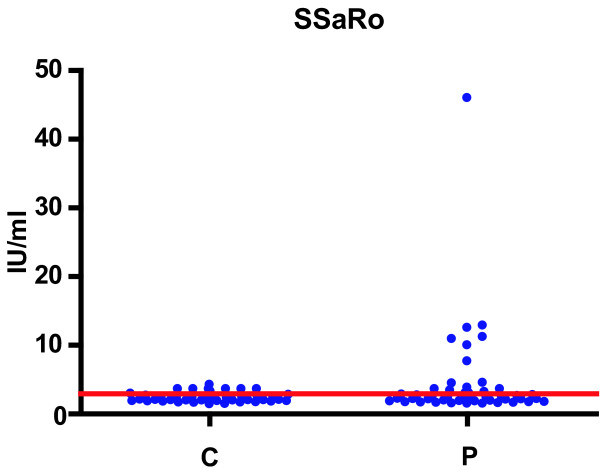
The anti-Ro/SSA serum IgG immunoreactivity of healthy controls and of patients with myeloma.

Cut off values of anti-Ro/SSA IgG reactivity obtained analyzing 50 healthy sera was 3.59 IU/ml. The enhanced IgG immunoreactivity to RoSSÀ antigen found in this work in 9/47 analyzed MM patients' sera (Fig. 3). These anti-RoSSÀ IgG positive patients were: 2 with IgA, 5 with IgG, and 2 without M component.

## Discussion

In this work, the immunoreactivity to one of the food constituents is found in some MM patients. It is manifested through the enhanced levels of serum IgA immunoreactivity to gliadin. Data obtained by ELISA tests indicate that especially anti-gliadin, IgA immunity is developed in patients with MM. It seems that the elevated immunoreactivities to gliadin could not be the consequence of overproduction of immmunoglobulins from M component, because the enhanced antigliadin IgA immunoreactivity was found in the serum of some of MM patients without M component as well as in patients with IgG myeloma too. It is also possible that in some patients serum antigliadin immunoreactivities could be hidden in immune complexes in circulation (CIC) and in these cases this reactivity could not be revealed by ELISA test. One of the solutions for this problem could be the electrophoresis of serum proteins performed in the alkaline conditions pH = 8.6 i.e., in conditions which enables dissociation of antibodies from antigen in CIC [9,10]; these antibodies physically separated from the gliadin by electrophoresis could then again react with gliadin (0.2%, or 0.4% in 0.5% SDS) in the test for immunofixation [3].

It must be mentioned that only the level of anti tTG-2 IgA immunoreactivity in patients with myeloma was significantly higher than these obtained in healthy controls. Besides, our results show that autoimmune determinants as the enhanced immunity to tTG-2, and to Ro/SSA antigen, which are present in patients with gluten intolerance [4,5] and autoimmune diseases [8] respectively, are present in some patients with MM, too.

Immunoreactivity to some of these antigens was also found in few of healthy controls, indicating that the immunoreactivities to tTG or to Ro/SSA antigens, or to gliadin are not strictly specific for MM, but that they are only one part of the complex immunological disturbances found in this disease. Findings from this work are in accordance with the previously published reports [11-14] pointing to the partial immunological similarity between MM and celiac disease.

This work indicates that gliadin as food antigen could at least partially contribute to the plethora of immunological disturbances in some patients with MM. Due to the aminoacid sequence equality or high homology between α-gliadin and γ-gliadin and *C albicans*–hyphal wall protein 1 (HWP1), and with calreticulin, it is also possible that the determined immunoreactivity with gliadin is in part the consequence of the cross reactivity with mentioned proteins [15].

## Conclusion

Data from this work showed the existence of the enhanced serum immunoreactivity to gliadin, tTG-2 and Ro/SSA antigens in some patients with MM and set up the question whether MM is also on the list of malignant diseases which are associated with immunological aspect of gluten intolerance [16-19]. They call for the additional research to elucidate the clinical importance of both: the elevated humoral immunity to food antigens and to some autoantigens in patients with MM.

## Authors' contributions

ZJ designed the study, interpreted the data and wrote the manuscript. AK–R and IB have done the ELISA blood testing, collected the data, and organized the database, while DG performed the statistical analysis. SJ, SM and BM enrolled the patients with myeloma, revised critically the manuscript and added important points to the discussion, JR have done determination of the type of heavy and light immunoglobulin chains presented in the M component in the serum, by immunofixation. IS helped with ELISA serum testing. All authors approved the final draft of the manuscript.
